# Intraocular myofibroblastoma tumour of the ciliary body: a case report and literature review

**DOI:** 10.1186/s12886-022-02411-0

**Published:** 2022-05-02

**Authors:** He Yu, Caixin Zhang, Nianting Tong, Xiu Wang, Liangyu Wang, Huimin Gong, Xin Liu, Zhanyu Zhou

**Affiliations:** 1 Department of Ophthalmology, Qingdao Municipal Hospital, 5 Middle Donghai Road, Shinan District, Qingdao, Shandong China; 2 Department of Ophthalmology, Dalian No,3 People’s Hospital, No. 40 Qianshan Road,Ganjingzi District, Dalian, Liaoning, China; 3 Department of Pathology, Qingdao Municipal Hospital, No.5 Donghai Middle Road, Shinan District, Qingdao, Shandong China

**Keywords:** Inflammatory myofibroblastoma tumor, ciliary body, immunohistochemistry

## Abstract

**Background:**

Inflammatory Myofibroblastoma Tumors (IMTs) are extremely tumour rare in the intraocular.

**Case presentation:**

A ciliary body tumor was found under slit lamp biomicroscopy in a 55-year-old male first diagnosed with cataract. Then this patient underwent trans-sclera resection via partial lamellar sclerouvectomy and par plans vitrectomy to remove the mass. Hematoxylin and eosin (HE) staining and immunohistochemistry findings showed that the characteristics of the tumor were consistent with IMT.

**Conclusions:**

We reported a rare case of intraocular IMT, which is confirmed by H&E staining, and IHC positive staining for Vimentin, Desmin and ALK, while negative staining for SMA, S-100, ki-67, CK, CD68, and calponin.

## Background

Inflammatory myofibroblastic tumour (IMT) is a distinctive lesion composed of myofibroblastic spindle cells with a prominent inflammatory infiltrate of lymphocytes, eosinophils, and plasma cells [1]. IMT is rare, but is found most often in the lungs and can occur throughout the body [2]. However, there have been few reports of IMT in the eyes [3]. Here, we describe a 55-year-old man with an IMT of the ciliary body.

## Case presentation

**Fig. 1:**
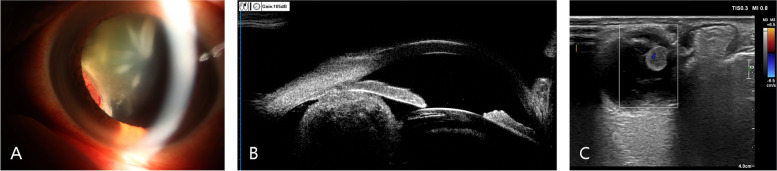
Under the slit lamp, superficial blood vessels can be seen on the surface, and the opacity of the lens is mainly located at the location of the mass. (**A**). Ultrasound biomicroscopy image show a solid mass of the ciliary body from 6:30 to 8:30 (**B**). The ultrasonography image shows a mass of about 0.5–0.6 cm in size with medium–high echo (**C**)

**Fig. 2:**
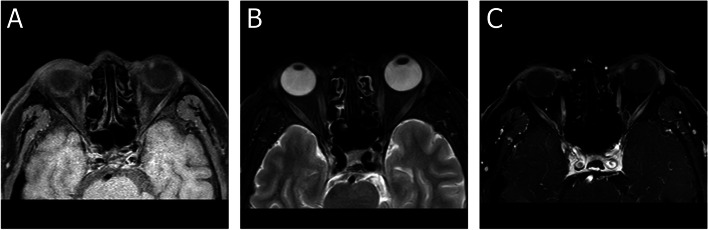
MRI of the eyeball and orbit. In the lens of the left eye, the signal intensity of the mass was slightly hyperintense on T1-weighted images (**A**) and markedly hypointense on T2-weighted images (**B**) and enhanced very well (**C**)

**Fig. 3:**
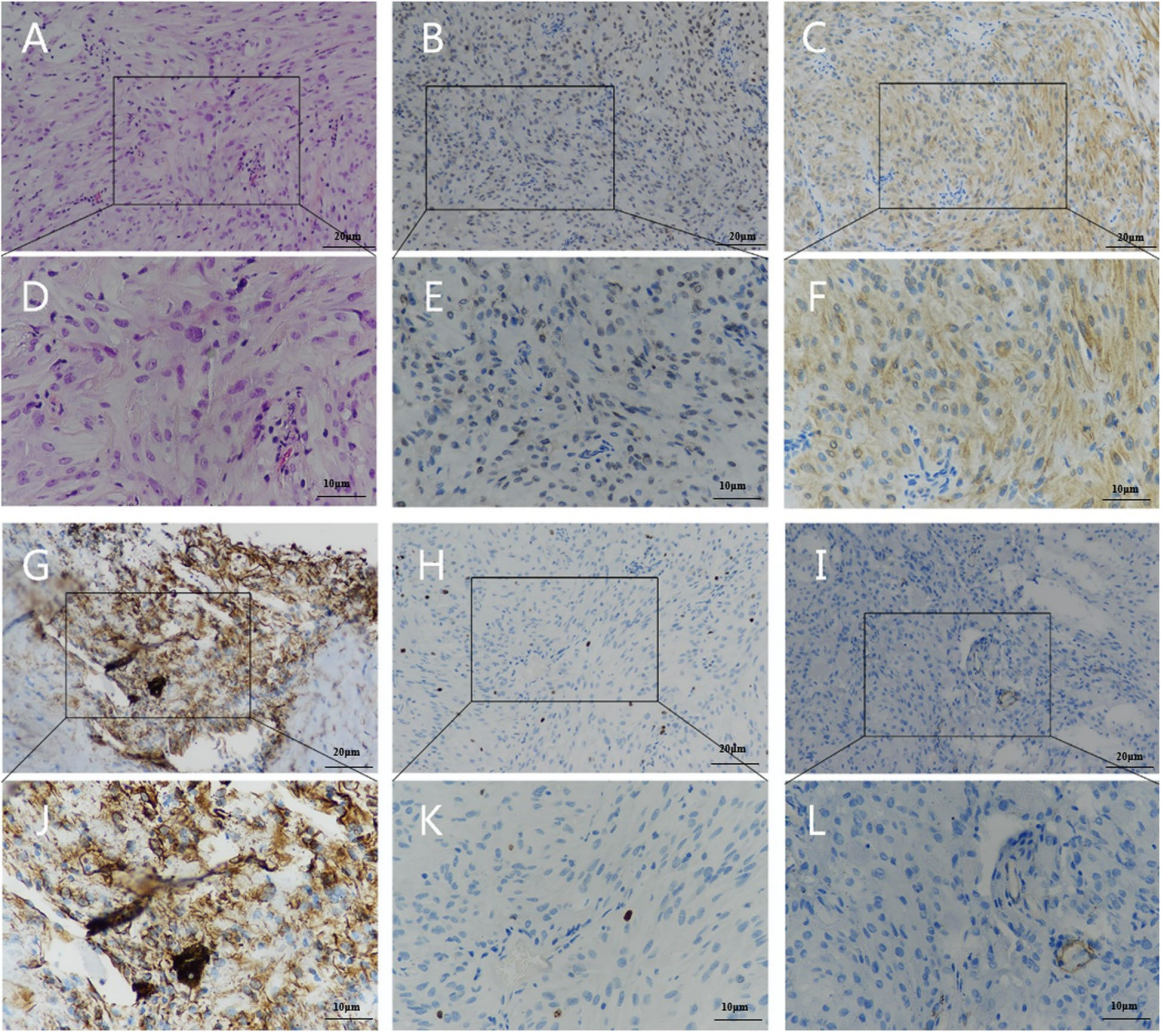
HE staining and IHC staining for ALK, Desmin, Vimentin, ki-67 and SMA. HE staining shows smooth muscle-like cells and some chronic inflammatory cell infiltration in the surrounding area(A 20 × ,D 40 ×) The positive staining images of ALK, Desmin and Vimentin are shown in (B 20 × ,E 40 ×), (C 20 × ,F 40 ×) and (G 20 × ,J 40 ×), respectively. While negative staining images of ki-67 and SMA are shown in (H 20 × ,K 40 ×) and (I 20 × ,L 40 ×), respectively

**Fig. 4:**
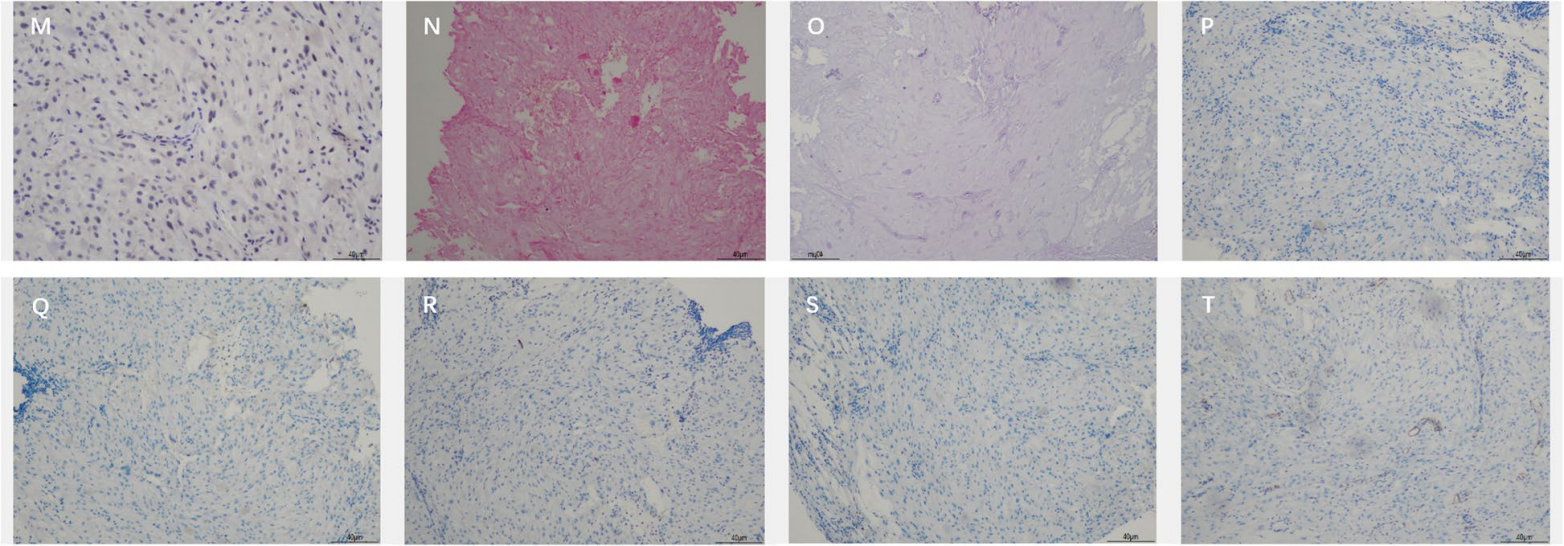
The patient’s p53 was wild-type (M 10X). The negative staining images of EBER、acid-fast staining、HHV-8、Melan-A、HMB-45、STAT and CD34 are shown in (N 10X) (O 10X) (P 10X) (Q 10X) (R 10X) (S 10X)and(T 10X), respectively. A scale bar is 40um on every image

A 55-year-old man presented with a chief complaint of progressive, painless vision loss in the left eye over the course of 20 days. The patient had no significant prior medical or ocular history. The patient had no history of smoking,had no previous trauma,and had no infected lesions.His best-corrected visual acuity (BCVA) and intraocular pressure (IOP) in the left eye were 20/100 and 15 mmHg, respectively. Only cortical opacity in the left lens was evident on slit-lamp biomicroscopy before pupil dilation. Therefore, an initial diagnosis of age-related cataract was made by outpatient physicians. The patient was then referred to a surgeon for cataract surgery. A dilated pupil examination under slit-lamp biomicroscopy revealed a well-defined, ovoid soft tissue amelanotic mass filling the inferonasal quadrants. Superficial vessels were evident on its surface and lens opacity was mainly present at the mass location (Fig. 1A). Ultrasound biomicroscopy examination revealed a solid mass in the ciliary body from 6:30 to 8:30 (Fig. 1B). Ultrasonographic examination of the tumour showed medium to high echodensity and an approximately 0.5–0.6-cm ovoid mass (Fig. 1C). Magnetic resonance imaging also revealed a well-marginated, ovoid soft tissue mass (0.5–0.6 cm). The mass was slightly hyperintense on T1-weighted images (Fig. 2A) and markedly hypointense on T2-weighted images (Fig. 2B), and demonstrated considerable enhancement (Fig. 2C). Based on these findings, the patient’s modified diagnosis was cataract in the left eye, secondary to ciliary body neoplasm. The neoplasm push againstthe lens and the lens is modified its metabolism, leading to lens opacity. Transillumination experiments were performed during the operation, and no transillumination defects were found. Surgical intervention was performed to investigate the pathological diagnosis, as well as to relieve symptoms and improve vision. Instead of enucleation, sclerotomy with tumour biopsy was performed because the tumour was considered benign. Trans-scleral resection was performed along with partial lamellar sclerouvectomy, pars plana vitrectomy, and silicon oil tamponade. The tumour was completely removed during the operation. Pathological examination was performed after complete removal of the tumour. Light microscopy(NIKON,Y-THM,made in JAPAN) showed that the tumour was composed of collagenised fibrous tissue. The tumour is a regular, amelanotic oval mass about 1 cm × 0.6 cm × 0.5 cm.Some regions contained substantial spindle cells with tumour-like proliferation, and chronic inflammatory cell infiltration in surrounding areas (Fig. 3A, D). Tumor cells are round to epithelioid, accompanied by vesicular nuclei, vacuolar chromatin and prominent nucleoli, and rich in interstitial mucus-like with neutrophil infiltration. Spindle-shaped myofibroblasts are loosely arranged, surrounded by edema-like mucus-like background, with a large number of blood vessels and inflammatory cells,plasma cells, lymphocytes, and eosinophils. No clear zonal maturation was seen under the microscope. The cells are spindle-shaped or oval. Nuclear atypia was not obvious. Nucleoli are not prominent. Lymphocyte infiltration was scattered around, and more lymphocytes aggregated around small blood vessels. The proliferation index of tumor cells is 2%(ki-67 2%). Immunohistochemical analysis revealed positive staining results for anaplastic lymphoma kinase (ALK) (Fig. 3B, E), desmin (Fig. 3C, F), and vimentin (Fig. 3G, J), as well as negative staining results for smooth muscle actin (SMA) (Fig. 3H, K), S-100, Ki-67 (Fig. 3I, L), CK, CD68, and calponin. Additional immunohistochemical experiments were performed for the patient, and the patient’s p53 was wild-type (Fig. 4M).

The final diagnosis based on histological and immunohistochemical assessments was IMT of the ciliary body.

The patient was followed up regularly after discharge, and the condition of body and eyes was stable. The silicone oil removal operation was performed in our hospital, and the operation went well.

## Discussion and conclusions

IMTs are mesenchymal neoplasms of intermediate biological potential with a predilection for the lung, abdomen, and pelvis [4]. Metastasis is rare in IMTs. Currently, IMTs are regarded as intermediate, locally recurrent, rarely metastasising neoplasms [5, 6].

IMTs are most have been observed in the orbit but extremely rare in the intraocular. Intraocular IMTs presumably arise from uveal tissues and are associated with uveitis [7]. Common manifestations include proptosis, ptosis, and painless loss of vision [3]. Dermarkarian et al. [8] suspected that these tumours were prone to invasive growth and recurrence, and therefore recommended complete removal as the first-line treatment. The diagnosis of IMTs may be delayed because of nonspecific presenting symptoms [9]. Definitive diagnosis of IMTs relies on histopathological examination and immunohistochemical characterisation. The histopathology of IMTs involves varying degrees of acute and chronic inflammation and fibrosis [10]. The principal components are plasma and spindle-shaped cells. The plasma cells are scattered throughout the lesions, often in small clusters. These plasma cells are mature, occasionally large, and multinucleated [2]. IMTs express vimentin (99%) and desmin (69%) [1]. Myofibroblasts express intermediate filaments and exhibit the VD phenotype, characterised by the presence of vimentin and desmin [11]. ALK expression has been documented in patients with IMT [12]. These tumours do not express myoglobin, cytokeratin, KP-1, CD30, or oestrogen receptor [13]. They can be difficult to distinguish from spindle cell tumours. The main differential diagnoses in the eye are leiomyomas、schwannoma and anaplastic large cell lymphomas (ALCLs).

Schwannoma is a begin proliferation of Schwann cells that encase the peripheral nerves [14].Histopathological characteristics of schwannoma: Antoni A: The tumor cells are arranged in a parallel strip or spiral shape. The nucleus is narrower at one end and can be arranged in a fence shape. Antoni B: The tumor tissue structure is loose, and the cells are arranged in vacuoles or nets. The tumor cells are lymphoid or star-shaped, relatively small in size, cytoplasmic protrusions are connected to each other in a network shape [15].

Leiomyomas are rare benign tumours, most commonly found in the uterus and alimentary tract [16]. On light microscopy analysis, the tumours are typically composed of interlacing bundles of spindle cells with blunt-ended oval nuclei, moderate amounts of eosinophilic fibrils in the cytoplasm, and intracellular myoglial fibrils [17]. Leiomyomas consistently express SMA, but do not tend to express ALK, S-100, or HMB-45 [18]. The tumour in our patient occurred in the ciliary body and spindle-shaped cells were revealed by haematoxylin–eosin staining. However, immunohistochemistry results were negative for SMA and ALK. Furthermore, the patient had chronic inflammatory cell infiltration, so a diagnosis of leiomyoma was not considered.

ALK expression is not a specific manifestation of IMT. Some aspects of the ALK activation mechanism in IMT are also present in a subset of ALCLs [1]. On light microscopy analysis, ALCLs are typically composed of interwoven fascicles of histiocytes and myofibroblasts combined with chronic inflammatory cells (e.g. T lymphocytes) [19]. The tumour in our patient was not regarded as ALCL, because of the large proportions of collagenised fibrous tissue and smooth muscle-like cells. Our patient had a Ki-67 level of 2%, indicating an absence of cell proliferation and thus excluding the possibility of ALCL. Notably, primary intraocular lymphomas are mostly large B-cell lymphomas; few originate from T cells [20].

After perfecting the relevant immunohistochemistry, it was found that EBER (Fig. 4N)was negative, acid-fast staining(Fig. 4O) was negative, and HHV-8 (Fig. 4P)was negative, so EBV, TB, and HHV-8 infection were excluded. It was found that Melan-A (Fig. 4Q)was negative, HMB-45(Fig. 4R) was negative, STAT(Fig. 4S) was negative, and CD34 (Fig. 4T)was negative.Therefore, melanoma and SFT can be excluded.

In our patient, other possible tumours were excluded on the basis of haematoxylin–eosin staining and immunohistochemical analysis. Therefore, a diagnosis of primary intraocular IMT tumour with ALK overexpression was considered.

Cytogenetic and molecular interpretations are important in this pathology. Molecular genetics of this type of tumor have previously described about 50% of cases with 2p23 (ALK) rearrangement, partner genes including ATIC, TPM3, TPM4, CLTC, etc. ALK-RANBP2 gene rearrangement is found in most epithelioid inflammatory myofibroblastic sarcomas. There are reports. Tumors negative for ALK gene fusions can have other fusions including ROS1, RET, etc.

However, there were several differences between our patient and the findings of previous reports on IMT. Generally, IMTs exhibit SMA expression (92%), because the most common intermediate filament feature expressed by myofibroblasts is the VA phenotype (i.e. expression of vimentin and α-SMA). However, there have been reports of IMTs with the VD phenotype [11]. Our patient’s clinical characteristics were consistent with the VD phenotype. ALK expression has been documented in 35–60% of patients with IMTs [12]. ALK markers are mainly characterised by light to moderate cytoplasmic staining. In addition to classic IMTs, there have been reports of special epithelioid subtypes [21, 22]. There are few reports of intraocular IMTs, so other subtypes of IMTs may exist with ALK staining in the nucleus.

In conclusion, we have described a rare instance of intraocular IMT, confirmed by haematoxylin–eosin staining, as well as positive immunohistochemical staining results for vimentin, desmin, and ALK. This patient had negative immunohistochemical staining results for SMA, S-100, Ki-67, CK, CD68, and calponin. Medline search did not reveal any report of IMT of the ciliary body.

## Data Availability

All data generated or analysed during this study are included in this published article [and its supplementary information files].

## Abbreviations

IMT: Inflammatory myofibroblastic tumor
BVCA: Best corrected visual acuity
IOP: Intraocular pressure
UBM: Ultrasound biological microscope
MRI: Magnetic resonance imaging
ALK: Anaplastic lymphoma kinase
SMA: Smooth muscle actin
ALCLs: Anaplastic large cell lymphomas
